# *Faecalibacterium prausnitzii* Supplementation Prevents Intestinal Barrier Injury and Gut Microflora Dysbiosis Induced by Sleep Deprivation

**DOI:** 10.3390/nu16081100

**Published:** 2024-04-09

**Authors:** Xintong Wang, Yixuan Li, Xifan Wang, Ran Wang, Yanling Hao, Fazheng Ren, Pengjie Wang, Bing Fang

**Affiliations:** 1 Key Laboratory of Precision Nutrition and Food Quality, Department of Nutrition and Health, China Agricultural University, Beijing 100083, China; xtwang@cau.edu.cn (X.W.); liyixuan@cau.edu.cn (Y.L.); wangran@cau.edu.cn (R.W.); haoyl@cau.edu.cn (Y.H.); renfazheng@cau.edu.cn (F.R.); 2 Department of Obstetrics and Gynecology, Columbia University, New York, NY 10032, USA; wangxfan@126.com; 3 Food Laboratory of Zhongyuan, Luohe 462000, China

**Keywords:** sleep deprivation, *Faecalibacterium prausnitzii*, intestinal barrier function, intestinal microflora, short chain fatty acids

## Abstract

Sleep deprivation (SD) leads to impaired intestinal barrier function and intestinal flora disorder, especially a reduction in the abundance of the next generation of probiotic *Faecalibacterium prausnitzii* (*F. prausnitzii*). However, it remains largely unclear whether *F. prausnitzii* can ameliorate SD-induced intestinal barrier damage. A 72 h SD mouse model was used in this research, with or without the addition of *F. prausnitzii*. The findings indicated that pre-colonization with *F. prausnitzii* could protect against tissue damage from SD, enhance goblet cell count and MUC2 levels in the colon, boost tight-junction protein expression, decrease macrophage infiltration, suppress pro-inflammatory cytokine expression, and reduce apoptosis. We found that the presence of *F. prausnitzii* helped to balance the gut microbiota in SD mice by reducing harmful bacteria like *Klebsiella* and *Staphylococcus*, while increasing beneficial bacteria such as *Akkermansia*. Ion chromatography analysis revealed that *F. prausnitzii* pretreatment increased the fecal butyrate level in SD mice. Overall, these results suggested that incorporating *F. prausnitzii* could help reduce gut damage caused by SD, potentially by enhancing the intestinal barrier and balancing gut microflora. This provides a foundation for utilizing probiotics to protect against intestinal illnesses.

## 1. Introduction

Sleep is an essential physiological process of the body, but with the acceleration of social rhythm, there is still a 1/3 of the world’s population that suffer from the insufficient sleep phenomenon [1]. Sleep loss can damage the body’s cognitive function, gastrointestinal digestion, absorption function and endocrine homeostasis [2,3,4]. Recently, research has found that sleep deprivation (SD) induces intestinal barrier dysfunction [5]; the prevention of disease-causing substances and bacteria from entering the bloodstream is the primary function of the intestinal mucosal barrier [6]. MUC2, the main mucin that forms gels in the intestines, plays a key role in creating the mucus barrier [7]. Tight-junction proteins like claudins, zona occludens, and occludin are responsible for preserving the integrity of the intestinal epithelial barrier [8]. The weakening of the intestinal epithelial barrier is marked by a reduction in mucous layer thickness, disturbance in the distribution of tight-junction proteins, and compromised intestinal integrity resulting in heightened intestinal permeability. This allows toxins to move into the lamina propria, triggering an exaggerated immune response from the host’s immune cells [9]. Since SD can seriously affect the intestinal health of the body, it is of great significance and great demand to find a healthy and green substance or method to alleviate the adverse effects of insomnia.

Recently, more and more studies have shown that severe intestinal flora imbalances are related to SD, so the research and application of microecological agents are receiving more attention [10]. Previous research has shown that SD can disrupt the balance of intestinal microorganisms in mice, resulting in a reduction in beneficial bacteria like *Faecalibacterium* [11]. When the intestinal barrier becomes more permeable, it is unable to effectively block out the microorganisms in the surroundings [12]. This leads to a significant amount of detrimental bacteria and their byproducts passing through the intestinal barrier during times of stress, subsequently impacting intestinal epithelial cells and immune cells in a chain reaction [13]. Nevertheless, certain research has indicated that adjusting and replenishing the equilibrium of gut bacteria could improve gut health and decrease long-lasting inflammation [14,15]. Therefore, preserving the balance of gut bacteria and improving the strength of the intestinal barrier could offer a novel approach to preventing and treating intestinal damage caused by SD.

*Faecalibacterium* is recognized as a key bacterium in maintaining a healthy gut, and fluctuations in its levels may indicate an imbalance in the human gut microbiota, making it a potential contender for future probiotics [16]. In patients with inflammatory bowel disease (IBD), there is a reduction in the richness of two mucosa-associated *Faecalibacterium* phylotypes [17]. In a recent study using multicenter amplicon sequencing data, Chen et al. found that, within a cohort of 708 individuals (354 irritable bowel syndrome (IBS) patients and 354 healthy controls), the genus *Faecalibacterium* was one of the depleted taxa in IBS patients [18]. Furthermore, *Faecalibacterium* abundance was found to be higher in healthy individuals than in individuals with mild Alzheimer’s disease patients, in whom it was positively correlated with cognitive performance [19]. It can be seen that *Faecalibacterium* is associated with a variety of human diseases. SCFAs are among the most thoroughly investigated bacterial metabolites; *Faecalibacterium* is considered the main producer of butyrate [20]. Butyrate serves as a crucial energy source for cells in the intestines and is essential for regulating intestinal peristalsis, maintaining epithelial barrier function, and supporting the immune system’s health [21,22]. In addition, butyrate is involved in the enhancement of the colon barrier by increasing the synthesis of tight-junction proteins and the production of antimicrobial peptides [23]. In chronic kidney disease, *Faecalibacterium* could restore the disturbed intestinal microflora and drive its metabolite butyrate to play renal protection [24]. The potential of *Faecalibacterium* to enhance intestinal health in sleep-deprived mice is uncertain, and further research is needed to understand the mechanism behind its effects.

In this study, we constructed an acute SD model for 72 consecutive hours and administered *Faecalibacterium prausnitzii* (*F. prausnitzii*) with gavage before SD. We analyzed the intestinal flora and intestinal barrier in mice through the identification of colon inflammation markers, LPS, proteins related to tight junctions, and 16s rDNA sequencing. Our results showed that *F. prausnitzii* could improve intestinal barrier damage and intestinal microflora disturbance induced by SD. Its metabolite butyrate has participated in the improvement of *F. prausnitzii* on intestinal damage.

## 2. Materials and Methods

### 2.1. Animal and Experimental Design

Forty-eight male ICR mice, 8 weeks old, from Vital River Laboratory Animal Technology Co., Ltd. in Beijing, China, were housed in cages under standard environmental conditions with a temperature of 21 ± 1 °C and relative humidity of 50 ± 10%. All animal experiments were performed at the SPF animal room, China Agricultural University. They were kept on a 14 h light/10 h dark cycle, with lights on at 7:00 a.m. and off at 9:00 p.m. The mice were provided with ad libitum access to food and water. All mice used in the experiments were housed in groups of four per cage under the same environmental and husbandry conditions. The mice were randomly divided into four groups: (1) control group (CON, *n* = 12); (2) sleep deprivation group (SD, *n* = 12); (3) *F. prausnitzii* colonization group (FP group, *n* = 12); and (4) sleep deprivation with *F. prausnitzii* colonization group (SD + FP group, *n* = 12). The number of mice for each group was determined based on similar experiments from the literature [25].

To construct a pseudo-sterile mouse model, all mice were given water with 1 g/kg ampicillin (A102048, Aladdin, Shanghai, China), 100 mg/kg gentamicin (G100391, Aladdin, Shanghai, China), 0.5 g/kg neomycin (N412785, Aladdin, Shanghai, China), 0.5 g/kg vancomycin (V105495, Aladdin, Shanghai, China), and 10 mg/kg erythromycin (E105345, Aladdin, Shanghai, China) for 10 days. A new solution of antibiotics was made daily to ensure its effectiveness. Mice (FP and SD + FP) received an oral gavage of 10^8^ CFU of *F. prausnitzii* (in 0.2 mL PBS) at 8 a.m. The CON and SD groups received a 0.2 mL PBS vehicle via oral gavage. After 14 days of inoculation, mice in the SD group and SD + FP group were subjected to SD for 72 h. A modified multi-platform water environment method was utilized to establish the SD mouse model [11]. Four mice were housed in one cage, and their body weight was recorded daily.

At 8 a.m. on the day the experiment concluded, every mouse was euthanized through anesthesia with chloral hydrate. The mouse colon contents were gathered and frozen at −80 °C for microbial analysis. One part of the colon was preserved in 4% paraformaldehyde for morphological examination, while another section was stored at −80 °C for RT-PCR and ELISA testing.

### 2.2. Probiotic Culture

The probiotic *F. prausnitzii* (DSM 17677) was obtained from DSMZ. *F. prausnitzii* probiotics were introduced into the Modified Reinforced Clostridial Broth Medium (MZMD039B, Ningbo Mingzhou Biotechnology Co., Ltd., Ningbo, China) and incubated at 37 °C for 48 h in an anaerobic setting. After centrifuging the probiotic culture, the supernatant was removed and the bacterial precipitates were then mixed with 20% glycerol. Plate counting was used to determine the bacterial content, and the bacterial solution was diluted to a final concentration of 10^8^ CFU (0.2 mL). Mice were treated via oral gavage with 200 μL of either *F. prausnitzii* suspension or anaerobic PBS.

### 2.3. Hematoxylin and Eosin (H&E) Staining

Freshly isolated colon samples were fixed with 4% paraformaldehyde overnight. After dehydration, the tissues were encased in paraffin and cut into slices that were 5 μm thick. Colon tissue sections were stained with H&E (G1120, Solarbio, Beijing, China) and evaluated histologically based on established criteria from a previous publication [26]. Each tissue section was microscopically assessed for the loss of epithelial surface, destruction of crypt and infiltration of immunocytes (each category scored from 0 to 4). The total scores indicate the cumulative pathological score recorded for every individual sample.

### 2.4. AB-PAS Staining

Colon tissue sections were treated with alcian blue and periodic acid-Schiff (AB-PAS) stains following the instructions provided by the manufacturer (G1285, Solarbio, Beijing, China). The average number of goblet cells in 30 randomly selected intact crypts per group was determined by calculating goblet cell counts.

### 2.5. Immunohistochemical Staining

Paraffin sections were treated with rabbit anti-ZO-1 (1:200, 21773-1-AP, Proteintech, Chicago, IL, USA), rabbit anti-mucin-2 (MUC2) (1:2000, 27675-1-AP, Proteintech, Chicago, IL, USA), rabbit anti-occludin (1:700, GB111401-100, Servicebio, Wuhan, China), rabbit anti-cleaved caspase-3 (1:200, 9661, CST, Boston, MA, USA), and mouse anti-F4/80 (1:1000, GB11027, Servicebio, Wuhan, China) primary antibodies overnight at 4 °C. Afterward, the portions were washed with 0.01M PBS (pH 7.4) and then exposed to biotinylated goat anti-rabbit IgG (1:300, GB23303, Servicebio, Wuhan, China) or rabbit anti-mouse IgG (1:300, GB23301, Servicebio, Wuhan, China) for a duration of 2 h at ambient temperature. Afterward, the portions were dyed with hematoxylin and placed on a mount. In every instance, slides lacking the main antibody were analyzed for comparison. Cells showing immunoreactivity exhibited a yellow-brown stain in their cytoplasm. Positive cells in five cross-sections were randomly selected for each sample, and at least 30 fields were counted for each group. The integrated optical density (IOD) was measured by using ImageJ software (version 4.0.2; Scion Corp., Frederick, MD, USA)

### 2.6. Real-Time Reverse Transcription-Polymerase Chain Reaction (RT-PCR)

Six colon samples were used to extract total RNA with the FastPure^®^ Cell/Tissue Total RNA Isolation Kit V2 (RC112; Vazyme, Nanjing, China). The HiScript III All-in-one RT SuperMix Perfect for qPCR (R333; Vazyme, Nanjing, China) was utilized to synthesize the initial cDNA strand. The ChamQ Universal SYBR qPCR Master Mix (Q711; Vazyme, Nanjing, China) was utilized for RT-PCR amplification. Every sample underwent two rounds of testing. Supplementary Table S1 contains the list of RT-PCR primers.

### 2.7. Enzyme-Linked Immunosorbent Assay (ELISA)

The colon tissues were collected for the detection of inflammatory factors, such as tumor necrosis factor alpha (TNF-α) (MK2868A; Meike, Yancheng, China) and interleukin-6 (IL-6) (MK5737A; Meike, Yancheng, China), and lipopolysaccharide (LPS) (MK3418A; Meike, Yancheng, China) concentrations using a competitive ELISA. The tests were conducted in compliance with the guidelines provided by the manufacturer. Every sample underwent two rounds of testing. Both the intra-assay and inter-assay coefficients of variation (CV) were below 15%.

### 2.8. Gut Microbiota Analysis

Samples of feces were gathered and frozen at −80 degrees Celsius for analysis using high-throughput DNA sequencing. Mouse feces were used to extract total DNA with the FastDNATM SPIN Kit from MP Bio in the Irvine, CA, USA. The Thermo Scientific Nanodrop 2000 (Waltham, MA, USA) was utilized to assess the strength and cleanliness of the samples. PCR was used to amplify DNA templates with specific primers 338F (ACTCCTACGGGAGGCAGCAG) and 806R (GGACTACHVGGGTWTCTAAT). PCR products were identified using 2% agarose gel electrophoresis, and were then purified and quantified using the qubit 4.0 system (Thermo Fisher Scientific, Waltham, MA, USA). Per the standard protocol of Majorbio Bio-Pharm Technology Co., Ltd. in Shanghai, China, the amplicons were combined in equal amounts for sequencing on the Illumina MiSeq PE300 platform. The analysis of data was conducted on the Majorbio cloud platform (cloud.majorbio.com, accessed on 6 November 2023).

### 2.9. Detection of SCFAs Using Ion Chromatography

Ion chromatography was used to determine the levels of short-chain fatty acids in feces as per the previously studied technique [27]. Briefly, around 25 mg of feces was mixed on a spin with 4 mL of sterile deionized water. Next, the specimen was spun at 8000 times the force of gravity at a temperature of 4 degrees Celsius for a duration of 10 min. After collection, the supernatant underwent filtration using a 0.22 mm mesh and was then analyzed for SCFAs content using an ion chromatography system (DIONEX ICS-3000, Thermo Fisher Scientific, Waltham, MA, USA).

### 2.10. Statistical Analysis

The information was presented as the average plus standard error and assessed with GraphPad Prism version 9 from GraphPad Software located in La Jolla, CA, USA. The data were analyzed with a normal distribution test and homogeneity test of variance. Group variances were compared using a one-way ANOVA, with Tukey’s post hoc test conducted afterwards. The Kruskal–Wallis test was used to examine variations in microbial composition among various groups at the genus level. Any *p*-values less than 0.05 were deemed to be statistically significant.

## 3. Results

### 3.1. F. prausnitzii Colonization Alleviated Intestinal Mucosal Barrier Disruption Induced by SD

**Figure 1:**
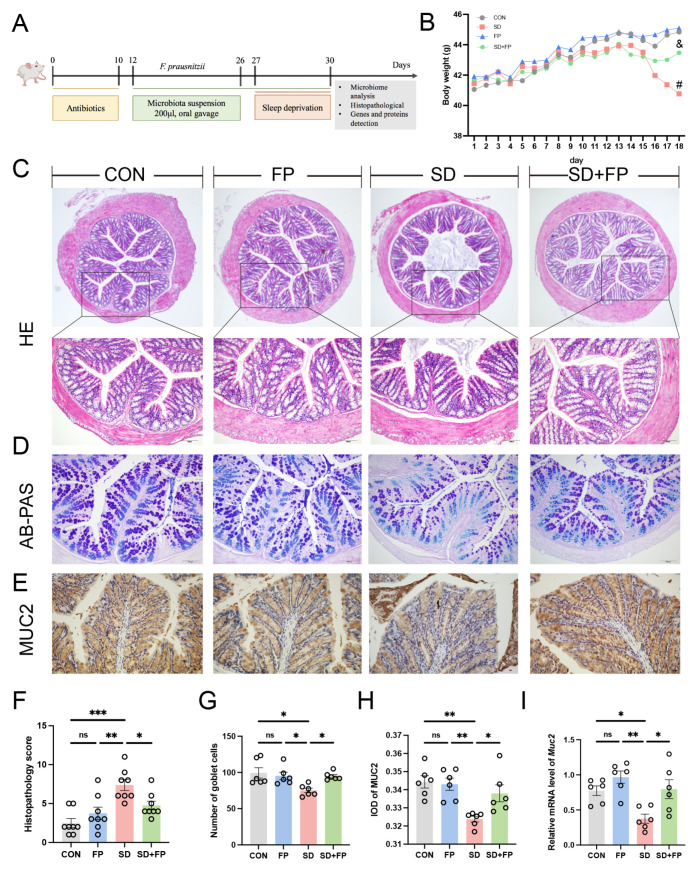
*Faecalibacterium prausnitzii* colonization alleviated intestinal mucosal barrier disruption induced by SD. (**A**) Diagram showing the layout of the experiment. (**B**) Body weight. (**C**) Hematoxylin and eosin (H&E) staining, Bar = 100 μm. (**D**) Alcian blue, and periodic acid-Schiff (AB-PAS) staining, Bar = 100 μm. (**E**) Representative captures of immunohistochemical of MUC2 in colon, Bar = 100 μm. (**F**) Histologic scores (*n* = 8). (**G**) The number of goblet cells per crypt (*n* = 6). (**H**) IOD of MUC2 in the intestinal tissue from each treatment group (*n* = 6). (**I**) The mRNA levels of Muc2 in the colon (*n* = 6). One-way ANOVA was utilized to evaluate variances. The study included a control group (CON), a group colonized with *Faecalibacterium prausnitzii* (FP), a group subjected to sleep deprivation (SD), and a group experiencing sleep deprivation with *Faecalibacterium prausnitzii* colonization (SD + FP). The outcome indicates the average value plus or minus the standard error. *, *p* < 0.05; **, *p* < 0.01; ***, *p* < 0.001 compared with the control group. ns, non-significance. # *p* < 0.05 vs. the CON group; & *p* < 0.05 vs. the SD group.

A mouse model was created to simulate intestinal barrier damage induced by SD, with or without colonization by *F. prausnitzii*, to investigate the potential beneficial effects of *F. prausnitzii* on the condition (Figure 1A). As illustrated in Figure 1B, we noticed a difference in the physical appearance of the mice, specifically in terms of their weight, across the CON, FP, SD, and SD + FP groups. Following a 72 h SD period, the weight of sleep-deprived mice was notably less than that of the control group. HE staining revealed that the control group exhibited normal tissues, while the SD group showed a breakdown of the epithelial barrier and invasion of inflammatory cells. However, pre-treatment with *F. prausnitzii* attenuated the above intestinal damage induced by SD (Figure 1C,F). Histological scoring revealed a significant increase in the SD group (66.1%, *p* < 0.001) compared to the CON group. However, colonization of *F. prausnitzii* alleviated the damage in the SD + FP group compared with the SD group (35.6%, *p* = 0.04).

AB-PAS staining was utilized to further identify the quantity of goblet cells. In the SD group, there was a notable decrease in the number of goblet cells in the colon (24.4%, *p* = 0.01) when compared to the control group (Figure 1D,G). Following the colonization of *F. prausnitzii*, there was a notable rise in the number of goblet cells in the colon within the SD + FP group (26.2%, *p* = 0.04) in contrast to the SD group (Figure 1D,G). MUC2, which is released by goblet cells, plays a significant role in safeguarding the intestinal epithelium. Immunohistochemistry results showed that the expression of MUC2 was significantly decreased in the SD group, compared with the CON group (5.9%, *p* = 0.002). In contrast, after *F. prausnitzii* colonization, the expression of MUC2 was significantly increased (4.4%, *p* = 0.04) in the SD + FP group compared with the SD group (Figure 1E,H). Additionally, it was discovered that the mRNA levels of Muc2 in the colon of sleep-deprived mice were decreased compared to the control mice (*p* = 0.03), but the colonization of *F. prausnitzii* significantly increased the expression of this gene (*p* = 0.02) (Figure 1I).

**Figure 2:**
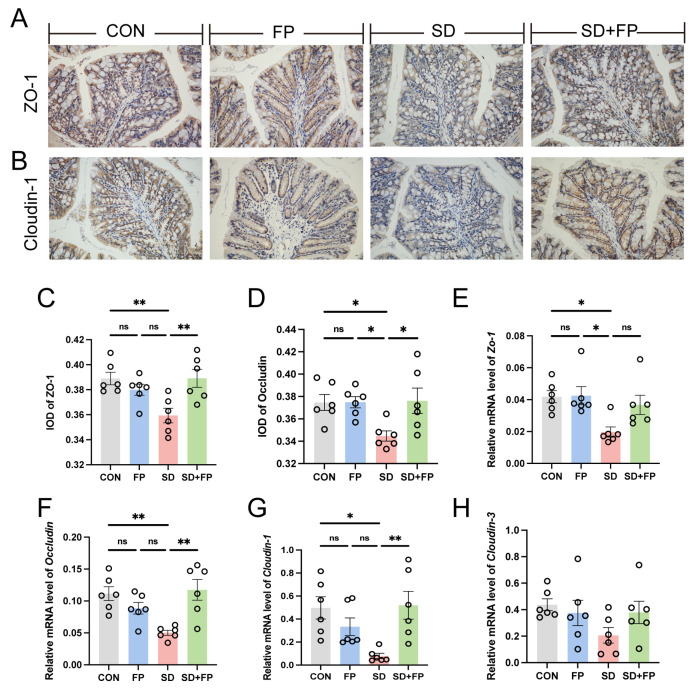
*Faecalibacterium prausnitzii* colonization increased the expression of tight-junction proteins in sleep-deprived mice. (**A**) Representative captures of immunohistochemical of ZO-1 in colon, Bar = 100 μm. (**B**) Representative captures of immunohistochemical of occludin in colon, Bar = 100 μm. (**C**) IOD of ZO-1 in the intestinal tissue from each treatment group (*n* = 6). (**D**) IOD of occludin in the intestinal tissue from each treatment group (*n* = 6). (**E**–**H**) The mRNA levels of *ZO-1*, *occludin*, *Cloudin-1*, and *Cloudin-3* in the colon (*n* = 6). One-way ANOVA was utilized to evaluate variances. The study included a control group (CON), a group colonized with *Faecalibacterium prausnitzii* (FP), a group subjected to sleep deprivation (SD), and a group experiencing sleep deprivation with *Faecalibacterium prausnitzii* colonization (SD + FP). The outcome indicates the average value plus or minus the standard error. *, *p* < 0.05; **, *p* < 0.01 compared with the control group. ns, non-significance.

Subsequently, the presence of tight-junction proteins was examined through immunohistochemistry and RT-PCR analyses. In the SD group, there was a significant decrease in the levels of ZO-1 (*p* = 0.008) and occludin (*p* = 0.05) tight-junction proteins compared to the CON group, as shown in Figure 2A–D. Conversely, following the colonization of *F. prausnitzii*, there was a notable increase in the expression of ZO-1 (*p* = 0.007) and occludin (*p* = 0.03) in the SD + FP group when compared to the SD group (Figure 2A–D). Furthermore, alterations in colonic tight-junction proteins were noted at the mRNA level in sleep-deprived mice. Specifically, the mRNA levels of colonic ZO-1, Cloudin-1, and occludin decreased after SD, but the presence of *F. prausnitzii* improved these markers (Figure 2E–G). Together, these data suggested that SD leads to the disruption of intestinal barrier integrity and decreased numbers of goblet cells but could be improved by *F. prausnitzii* colonization.

### 3.2. The Colonization of F. prausnitzii Suppressed the Production of Inflammatory Cytokines in SD Mice

**Figure 3:**
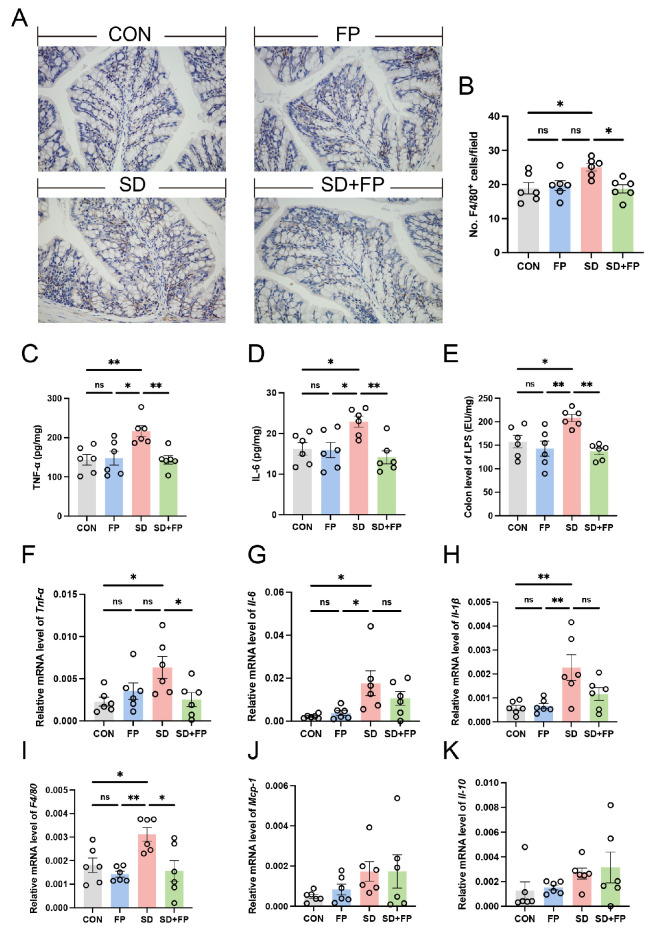
*Faecalibacterium prausnitzii* colonization inhibited the expression of inflammatory cytokines in sleep-deprived mice. (**A**) Representative captures of immunohistochemical of F4/80 in colon, Bar = 100 μm. (**B**) The quantification of F4/80+ cells (*n* = 6). (**C**,**D**) The levels of cytokines (TNF-α and IL-6) in the colon (*n* = 6). (**E**) The levels of LPS in the colon (*n* = 6). (**F**–**K**) The mRNA levels of Tnf-α, Il-6, Il-1β, F4/80, Mcp-1 and Il-10 in the colon (*n* = 6). One-way ANOVA was utilized to evaluate variances. The study included a control group (CON), a group colonized with *Faecalibacterium prausnitzii* (FP), a group subjected to sleep deprivation (SD), and a group experiencing sleep deprivation with *Faecalibacterium prausnitzii* colonization (SD + FP). The outcome indicates the average value plus or minus the standard error. *, *p* < 0.05; **, *p* < 0.01 compared with the control group. ns, non-significance.

We assessed alterations in inflammatory markers and macrophages in the colon to examine the inflammation response triggered by SD. By utilizing F4/80 as a marker for macrophages, we confirmed that there was a 32.6% increase in the amount of F4/80-positive cells in the colon of the SD group compared to the CON group (Figure 3A,B). We also observed a significant increase in IL-6 (41.3%, *p* = 0.04), and TNF-α (50.6%, *p* = 0.009) levels in the SD group compared with the CON group (Figure 3C,D). Nevertheless, the establishment of *F. prausnitzii* notably inhibited the stimulation of macrophages and reduced the levels of inflammatory molecules. Specifically, there was a decrease in the quantity of F4/80-positive cells in the colon of the SD + FP group compared to the SD group, with a significance level of *p* = 0.02. We also observed a significant decrease in IL-6 (38.3%, *p* = 0.005) and TNF-α (33.7%, *p* = 0.008) levels in sleep-deprived mice with *F. prausnitzii* colonization compared with SD mice. Additionally, the expression of pro-inflammatory cytokines (Tnf-α, Il-1β, Il-6) and F4/80 mRNA decreased in sleep-deprived mice following the introduction of *F. prausnitzii* (Figure 3F–I). Interestingly, we also observed a significant increase in LPS in the colon in the sleep-deprived mice versus the control mice, while the colonization of *F. prausnitzii* reversed this change (Figure 3E). These results suggested that the colonization of *F. prausnitzii* could improve the intestinal inflammation caused by SD.

### 3.3. Colonization of F. prausnitzii Mitigated Gut Microbiota Dysbiosis Induced by SD in Mice

**Figure 4:**
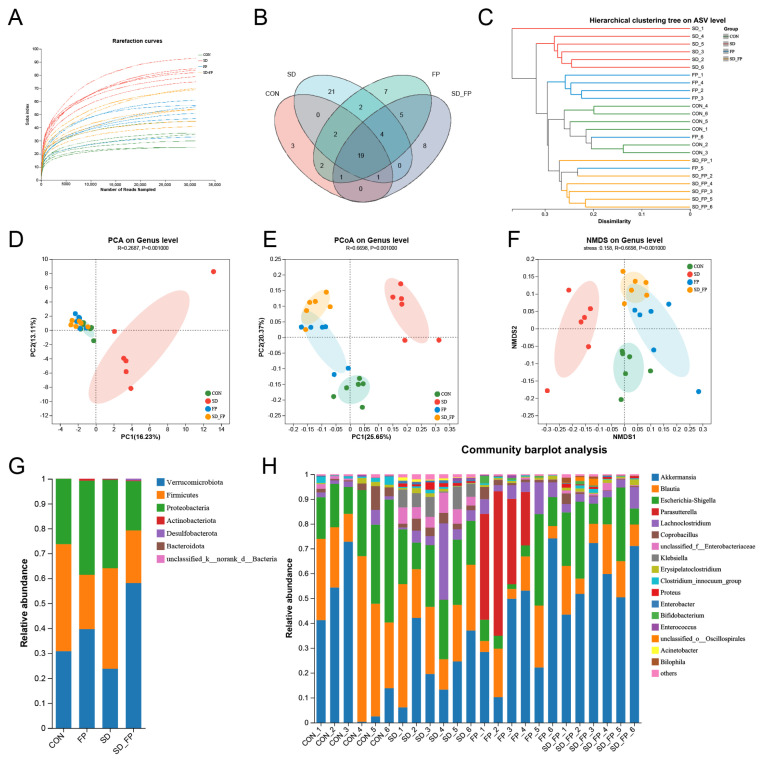
Composition of the colonic microbiota in mice. (**A**) Rarefaction curves. (**B**) Venn diagram. (**C**) Unweighted pair-group method with arithmetic mean (UPGMA) analysis (at the genus level). (**D**) Principal component analysis (PCA). (**E**) PCoA score plot. (**F**) Nonmetric multidimensional scaling (NMDS) score plot based on the binary_jaccard distance plot based on the ASV of the gut microbe. (**G**) Relative abundances of gut microbiota at the phylum level. (**H**) Relative abundances of gut microbiota at the genus level. The study included a control group (CON), a group colonized with *Faecalibacterium prausnitzii* (FP), a group subjected to sleep deprivation (SD), and a group experiencing sleep deprivation with *Faecalibacterium prausnitzii* colonization (SD + FP).

**Figure 5:**
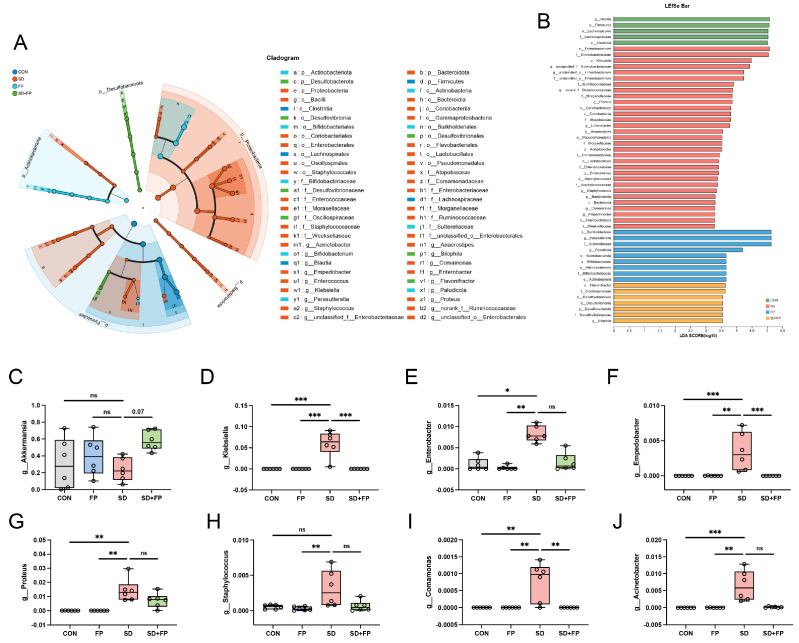
*Faecalibacterium prausnitzii* colonization inhibited the colonic microbial dysbiosis induced by sleep deprivation. (**A**) Taxonomic cladogram obtained from LEfSe sequence analysis in the colon. Biomarker taxa are highlighted by colored circles and shaded areas. (**B**) The diameter of each circle reflects the abundance of that taxon in the community. A cutoff value of 3 was used for LDA. (**C**–**J**) Relative abundance of *g__Akkermansia*, *g__Klebsiella*, *g__Enterobacter*, *g__Enterobacter*, *g__Proteus*, *g__Staphylococcus*, *g__Comamonas*, *g__Acinetobacter* in the colon microbiota based on the LefSe results. The study included a control group (CON), a group colonized with *Faecalibacterium prausnitzii* (FP), a group subjected to sleep deprivation (SD), and a group experiencing sleep deprivation with *Faecalibacterium prausnitzii* colonization (SD + FP). The outcome indicates the average value plus or minus the standard error. *, *p* < 0.05; **, *p* < 0.01; ***, *p* < 0.001 compared with the control group. ns, non-significance.

To examine how the presence of *F. prausnitzii* affects the composition of the gut microbiota in mice subjected to SD, 16s rDNA gene sequencing was performed. In total, 24 samples were collected from four groups of mice (*n* = 6) and subsequently sequenced to generate V3–V4 16s rDNA gene profiles. The Venn diagram indicated that SD treatment or *F. prausnitzii* colonization resulted in different microbial changes in mice (Figure 4B). β-diversity analysis measures the level of dissimilarity among various microbial communities (Figure 4D–F). The Bray–Curtis PCoA analysis indicated distinct group separation, with PC1, PC2, and PC3 explaining 25.65%, 20.37%, and 13.60% of the variation, respectively (Adonis, *p* = 0.001, R2 = 0.6698, Figure 4E). UPGMA analysis indicated that the SD + FP group exhibited a closer relationship with the CON group compared to the SD group, further supporting the findings of PCoA (Figure 4C). Verrucomicrobiota, Firmicutes, and Proteobacteria were the most common microorganisms at the phylum level (Figure 4G). At the genus level, *Akkermansia*, *Blautia*, *Escherichia-Shigella*, *Parasutterella*, *Lachnoclostridium*, *Coprobacillus*, and *Klebsiella* were the predominant floras (Figure 4H). LDA and LEfSe were used to determine the particular bacterial phyla linked to CON, FP, SD, and SD + FP groups, to pinpoint the key taxa that could account for the variations among the groups. Additionally, the LEfSe analysis revealed 52 taxa biomarkers in three groups, each identified with an LDA score greater than 3 and a *p*-value less than 0.05 (Figure 5A,B). *Akkermansia* was the most abundant bacterium at the genus level, and its content in the SD + FP group was the highest among all groups. Specifically, the relative abundance of *Akkermansia* was higher in SD + FP group than in SD group (*p* = 0.07, Figure 5C). Furthermore, the comparative prevalence of *Klebsiella* (*p* < 0.001, LDA score = 4.49, Figure 5D), *Enterobacter* (*p* = 0.002, LDA score = 3.78, Figure 5E), *Empedobacter* (*p* < 0.001, LDA score = 3.29, Figure 5F), *Proteus* (*p* < 0.001, LDA score = 3.86, Figure 5G), *Staphylococcus* (*p* = 0.01, LDA score = 3.35, Figure 5H), *Comamonas* (*p* < 0.001, LDA score = 3.29, Figure 5I), and *Acinetobacter* (*p* < 0.001, LDA score = 3.53, Figure 5J) was notably elevated in the SD group compared to the CON, FP, and SD + FP group (*p* < 0.05).

### 3.4. F. prausnitzii Colonization Alleviated Colon SCFAs Reduction in SD Mice

**Figure 6:**
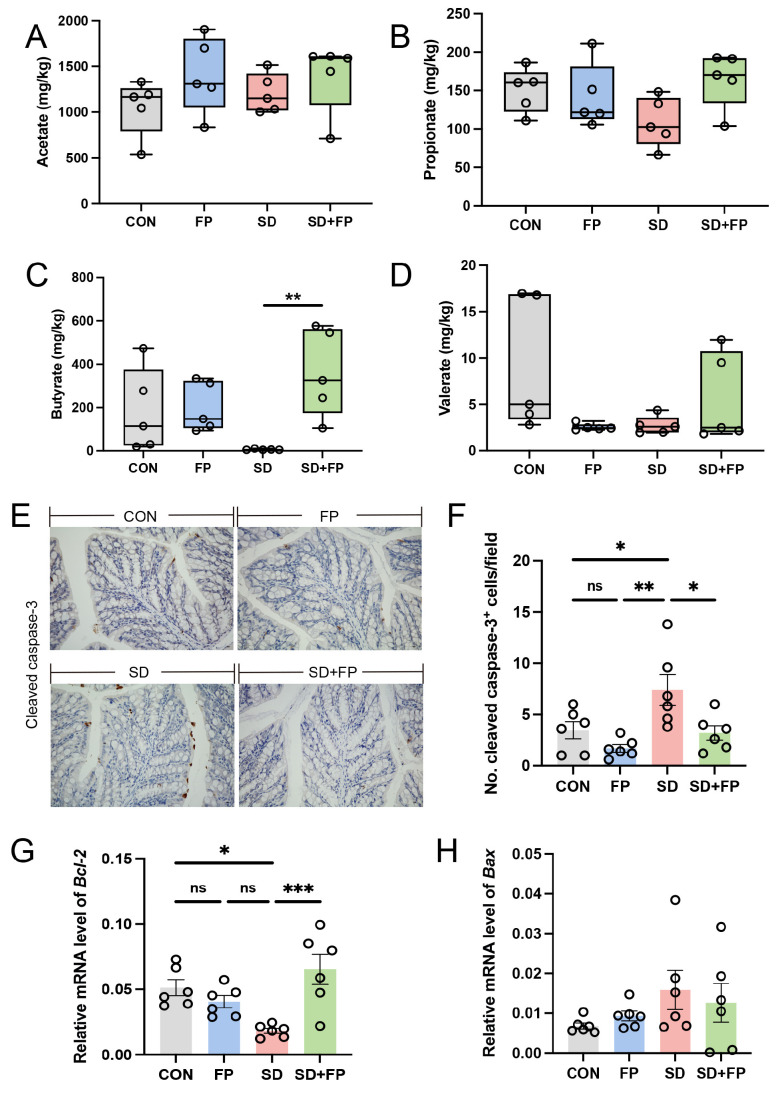
*Faecalibacterium prausnitzii* colonization reversed decrease in SCFAs and increase in intestinal apoptosis induced by sleep deprivation. (**A**) The colon acetate concentration (*n* = 5). (**B**) The colon propionate concentration (*n* = 5). (**C**) The colon butyrate concentration (*n* = 5). (**D**) The colon valerate concentration (*n* = 5). (**E**) Representative captures of immunohistochemical of cleaved caspase-3 in colon, Bar = 100 μm. (**F**) The quantification of cleaved caspase-3+ cells (*n* = 6). (**G**,**H**) The mRNA levels of Bax and Bcl-2 in the colon (*n* = 6). One-way ANOVA was utilized to evaluate variances. The study included a control group (CON), a group colonized with *Faecalibacterium prausnitzii* (FP), a group subjected to sleep deprivation (SD), and a group experiencing sleep deprivation with *Faecalibacterium prausnitzii* colonization (SD + FP). The outcome indicates the average value plus or minus the standard error. *, *p* < 0.05; **, *p* < 0.01; ***, *p* < 0.001 compared with the control group. ns, non-significance.

We assessed the concentrations of short-chain fatty acids in the feces of various treated mice using ion chromatography, as these are crucial metabolites produced by Faecalibacterium. The results showed that the level of fecal butyrate was decreased in the SD group compared with the CON group. In addition, the level of fecal butyrate was higher in the FP group than in the SD group. However, after colonization with *F. prausnitzii* in sleep-deprived mice, the level of fecal butyrate was significantly increased (*p* = 0.009) compared to the sleep-deprived mice (Figure 6C). No notable variances were observed in the levels of acetate in the feces (Figure 6A), propionate (Figure 6B), and valerate (Figure 6D) among the four groups (*p* > 0.05).

### 3.5. F. prausnitzii Colonization Reduced Intestinal Apoptosis Level Induced by SD

The inflammatory response is accompanied by the excessive apoptosis of cells engaged in inflammation. The SD group showed a notable increase in cleaved caspase-3 expression compared to the CON group (*p* = 0.04). However, the colonization with *F. prausnitzii* reversed these changes. The protein levels of cleaved caspase-3 were lower in the SD + FP group compared to the SD group, with a significance level of *p* = 0.03 (Figure 6E,F). In addition, we quantified the mRNA expression of pro-apoptotic Bax and anti-apoptotic Bcl-2. The findings indicated that SD decreased the expression of the anti-apoptotic protein Bcl-2, but colonization with *F. prausnitzii* reversed these effects (Figure 6G). No significant changes were detected for Bax levels in different treated groups (Figure 6H).

## 4. Discussion

**Figure 7:**
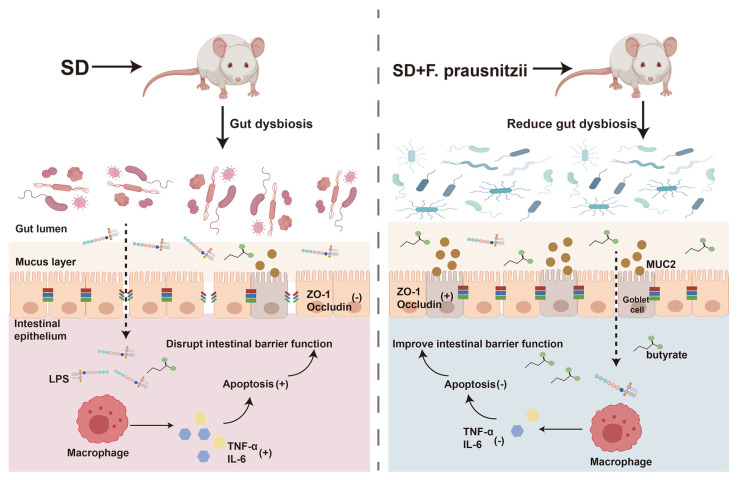
Schematic diagram of the potential mechanism by which *Faecalibacterium prausnitzii* ameliorates intestinal injury induced by sleep deprivation. A feasible mechanism is that the colonization of *Faecalibacterium prausnitzii* ameliorates impaired intestinal barrier function in SD mice through regulating intestinal inflammation, programmed death, and the gut microbiota. SD: sleep deprivation; LPS: lipopolysaccharide; ZO-1: tight-junction protein zonula occluden-1; TNF-α: tumor necrosis factor-alpha; IL-6; interleukin-6. (+); promote; (-); inhibit.

Sleep is the most essential physiological function of the human body, which is both important and complex. Lack of sleep can cause problems with the digestive system, immune system, metabolism, and circulation [28,29,30]. Our previous research has shown that SD could affect the intestinal barrier in mice, leading to changes in gut bacteria, including a decrease in beneficial *Faecalibacterium* in the colon [11]. *Faecalibacterium*, as the next generation of probiotics, has diverse functional effects such as anti-inflammatory effects, antioxidant and antimicrobial [31]. Furthermore, it was recently found that *Faecalibacterium* could ameliorate renal dysfunction in patients with chronic kidney disease partly through the butyrate-mediated GPR43 signaling in the kidney [24]. Uncertainty remains regarding the potential of *F. prausnitzii* colonization to repair intestinal barrier damage and restore intestinal microflora balance in sleep-deprived mice. In this study, by constructing an SD mouse model with *F. prausnitzii* intervention, it was found that SD caused intestinal microflora disturbance and intestinal permeability increase in mice, which then led to the introduction of toxic substances into the intestinal cavity to induce inflammation and the programmed death of colon epithelial cells. The preimplantation of *F. prausnitzii* remodeled the disturbed intestinal microflora in sleep-deprived mice, improved overtransition inflammation and apoptosis, enhanced intestinal integrity, and ultimately improved intestinal function (Figure 7).

Lack of sleep may impact the gut tissue in both structure and performance [32]. In this study, we found that acute SD caused the disruption of colonic epithelial cell morphology and function in mice. Through HE staining, it could be observed that there is inflammatory cell infiltration and intestinal epithelial cell damage in the colon of SD mice, and the colonization of *F. prausnitzii* could improve it. Goblet cells produce mucin glycoproteins that create a protective mucus barrier to prevent bacterial infiltration [33,34]. The main mucin is found in the intestines. The deletion of the MUC2 gene destroyed the intestinal mucus barrier and disturbed intestinal symbiotic flora, which made mice sensitive to the colonization of Citrobacter muris [35]. Goblet cells and MUC2 protein expression were significantly decreased in the SD group compared to the control group but were restored to normal levels with *F. prausnitzii* colonization. Maintaining the integrity of the intestinal epithelial cell barrier is dependent on the consistent presence of tight-junction protein. Exposure to stress or infection can disrupt the regulation of tight-junction protein expression in the body, leading to an increase in the size of the intestinal epithelial paracellular space. This can expose lamina propria immune cells to harmful bacteria, ultimately causing inflammation in the intestines [36,37]. A previous study found that the supernatant of *F. prausnitzii* enhances the intestinal barrier function by affecting paracellular permeability and may thereby attenuate the severity of DSS-induced colitis in mice [38]. In sleep-deprived mice, the levels of ZO-1, occludin, and Claudin-1, which are colonic proteins related to tight junctions, were found to be notably decreased. However, the presence of *F. prausnitzii* was able to bring back the expression of these tight-junction proteins.

Additionally, we assessed alterations in the intestinal microflora composition in the colon contents of mice following various treatments. Overall, the alpha-diversity and beta-diversity results showed that the microbiomes of the SD + FP group were similar to the CON group, while the SD group was less similar to the other groups, suggesting that the colonization of *F. prausnitzii* improved the disturbed intestinal microflora of SD mice. During the research, we noticed a rise in the proportion of *Klebsiella*, *Proteus*, *Staphylococcus*, and *Enterococcus* in sleep-deprived mice. *Klebsiella*, a prevalent pathogen, can be found in the respiratory system and gut of humans and can produce different harmful elements like adhesins, capsular polysaccharides, siderophores, and lipopolysaccharides to induce the body’s immune response [39]. A prior investigation discovered that *Klebsiella pneumoniae* triggered IBD by activating caspase-11-mediated IL18 in the cells lining the intestines [40]. *Proteus* is a common intestinal symbiotic bacterium, which is considered a potential pathogenic bacterium [41]. The relative abundance of *Proteus* was found to be significantly increased in MPTP-induced Parkinson’s disease [42]. *Staphylococcus* is a Gram-positive bacterium that could cause pneumonia, pseudomembranous colitis, pericarditis, sepsis, and other acute and chronic infections [43]. In general, we have observed the increase in various conditional bacteria in sleep-deprived mice. Conversely, following colonization by *F. prausnitzii*, there has been a notable reduction in the proportion of detrimental bacteria in the intestines of sleep-deprived mice. It shows that *F. prausnitzii* has corrected the disordered intestinal microflora caused by SD. Additionally, there was a notable rise in the proportion of *Akkermansia* in sleep-deprived mice following the colonization of *F. prausnitzii*. *Akkermansia* is a mucin-degrading bacteria in the gut. The addition of *Akkermansia* led to an increase in the quantity of goblet cells that produce mucin in the mice [44]. In addition, in the mucous layer, there are still some bacteria that cannot degrade mucus, but use carbon and nitrogen generated by mucin degradation by *Akkermansia*; these microorganisms include *F. prausnitzii*, *Rothella*, etc. Studies have shown that when Akkermansia and Faecalibacterium are co-cultured, they exhibit synergistic growth and produce butyrate [45]. Overall, our findings indicated that the presence of *F. prausnitzii* can restore the balance of gut bacteria in sleep-deprived mice by increasing the levels of beneficial bacteria and reducing the levels of harmful bacteria.

Additionally, there was a notable rise in the proportion of various detrimental microbes in the intestines of sleep-deprived mice, particularly *Klebsiella*. The research discovered that *klebsiella* pneumoniae can stimulate the generation of fully developed IL18 in colon epithelial cells and gut organoids, leading to colitis and enhancing DSS-induced colitis [40]. Following SD, our findings indicated a notable rise in the presence of LPS in the colon, along with an observed increase in gut permeability. Therefore, we speculated that the excess LPS produced by the increased harmful bacteria may cross the intestinal barrier and enter the lamina propria of the colon. TLR4 is a receptor for bacterial LPS in response to LPS-induced inflammation [46]. The activation of NF-κB leads to the production of different inflammatory mediators and cytokines [47,48]. Likewise, we noted that the presence of *F. prausnitzii* was able to decrease macrophage infiltration and lower the amount of pro-inflammatory cytokines in the colon. Similar to our results, *F. prausnitzii* generates butyrate to support Th17/Treg equilibrium and improve colorectal colitis by blocking histone deacetylase 1 [49]. Hence, restoring gut immune balance with Faecalibacterium could improve intestinal damage caused by SD.

*Faecalibacterium* is a major butyrate-producing bacteria and most of its functions are based on its metabolites. As a result, we also assessed the concentration of short-chain fatty acids present in the colon contents. Similar to our previous findings, the content of butyrate in the colon contents of sleep-deprived mice has decreased significantly [50]. Interestingly, the content of butyrate in sleep-deprived mice has increased significantly after pre-colonization with *Faecalibacterium*. It could be seen that the colonization of *Faecalibacterium* has significantly increased the content of butyrate, its metabolite. However, alterations in various other short-chain fatty acids (acetate, propionate, and valerate) did not show statistical significance among the different treatment groups. The research has shown that butyrate enhances the production of mucus in the goblet cells of the intestines, reinforces the chemical defense, and boosts the synthesis of tight-junction protein, which helps restore the compromised physical barrier [51]. Moreover, butyrate exhibits a beneficial anti-inflammatory impact on both intestinal epithelial cells and immune cells [52,53]. In prior research, it was noted that *Prevotellaceae* generates butyrate to reduce cardiotoxicity associated with PD-1/PD-L1 inhibitors through the PPARα-CYP4X1 pathway in macrophages located in the colon [54]. Hence, we hypothesized that the positive impact of *F. prausnitzii* colonization on intestinal barrier impairment in sleep-deprived mice is due to the significant generation of its metabolite butyrate. According to the previous results, we have observed the reduction in goblet cells, and the colonization of *F. prausnitzii* could alleviate them to a certain extent. Studies have shown that butyrate can regulate inflammatory response by inhibiting the NF-κB pathway, effectively inhibiting the apoptosis pathway, and ultimately improving intestinal barrier function [55]. Hence, we speculated that the presence of *F. prausnitzii* has a beneficial effect in decreasing colon cell apoptosis. In sleep-deprived mice, the colon exhibited elevated levels of cleaved caspase-3 protein and reduced levels of Bcl-2 anti-apoptotic factor mRNA, as indicated by our findings. Unlike sleep-deprived mice, no significant apoptosis has been observed in sleep-deprived mice colonized by *F. prausnitzii*.

Based on the worldwide human health problem of sleep loss, this study first explored the relationship between *F. prauznitzi* and sleep deficiency from the perspective of probiotics. In addition, this study evaluated the beneficial effects of colonization of *F. prauznitzi* on intestinal damage in sleep-deprived mice from multiple aspects of the intestinal barrier, including the mechanical barrier, immune barrier and microbial barrier. More importantly, through the detection of intestinal short-chain fatty acids, the relationship between *F. prauznitzii* and butyrate was found, which provided the direction for further exploration of the mechanism of *F. prauznitzii*. There are some limitations to our study. The animal model we constructed in this study was used to simulate acute SD, but a large number of people also face chronic sleep restriction in their daily lives. Therefore, in the follow-up research, it is necessary to expand the breadth of sleep research and more comprehensive research on the beneficial effects of *F. prauznitzii* on organismal health. In addition, the results of this study are based on animal models, and while our results provide valuable insights into the role of *F. prauznitzii* in alleviating intestinal damage in sleep-deprived mice, further research is needed to apply these results to human health.

*F. prauznitzii*, a health-related human intestinal bacterium with reduced levels in patients with a variety of metabolic diseases and IBD, is considered a next-generation probiotic with therapeutic potential. However, *F. prausnitzii* is strictly anaerobic, and two challenges need to be addressed before it can be used in human subjects. The first is that bacteria need to be produced on a large scale under strict anaerobic conditions, and the second is that adequate hypoxia conditions must be maintained throughout the culture process (such as centrifugation, filtration or lyophilization) [56]. At present, some studies have carried out preliminary exploration, using the symbiotic relationship between *F. prausnitzii* and *Desulfovibrio piger*, a sulfate-reducing bacterium, which can be used for the large-scale production of *F. prausnitzii* and improve storage stability [20]. Therefore, exploring the health effect of *F. prausnitzii* in the human body and transforming it for industry is the focus of future research. In addition, as scientists become more and more clear about the classification of *F. prausnitzii*, exploring and verifying the mechanism of action of *F. prausnitzii* in humans or animals is also the focus of future research.

## 5. Conclusions

The findings of our study indicated that *F. prausnitzii*, when used as probiotics, had a positive impact on alleviating the intestinal damage induced by SD. The positive impact of *F. prausnitzii* could be due to its ability to prevent SD-induced inflammation in the intestines, reduce dysbiosis in the gut microbiome, and promote the function of the intestinal barrier by potentially involving its metabolite butyrate in the process. These results provided new evidence for the protective mechanisms of *F. prausnitzii* against intestinal injury, thereby revealing the potential values of *F. prausnitzii* in SD-related body damage and having significant implications for *F. prausnitzii* as probiotics.

## Data Availability

Data are contained within the article.

## Supplementary Materials

The following supporting information can be downloaded at: https://www.mdpi.com/article/10.3390/nu16081100/s1, Table S1: Primers for real-time PCR.

## References

[1] Kroese F.M., Evers C., Adriaanse M.A., de Ridder D.T.D. (2016). Bedtime procrastination: A self-regulation perspective on sleep insufficiency in the general population. J. Health Psychol..

[2] Zamore Z., Veasey S.C. (2022). Neural consequences of chronic sleep disruption. Trends Neurosci..

[3] Neroni B., Evangelisti M., Radocchia G., Di Nardo G., Pantanella F., Villa M.P., Schippa S. (2021). Relationship between sleep disorders and gut dysbiosis: What affects what?. Sleep. Med..

[4] Zeng Y., Zhang Z., Liang S., Chang X., Qin R., Chen H., Guo L. (2023). Paternal sleep deprivation induces metabolic perturbations in male offspring via altered LRP5 DNA methylation of pancreatic islets. J. Pineal Res..

[5] Fang D., Xu T., Sun J., Shi J., Li F., Yin Y., Wang Z., Liu Y. (2023). Nicotinamide Mononucleotide Ameliorates Sleep Deprivation-Induced Gut Microbiota Dysbiosis and Restores Colonization Resistance against Intestinal Infections. Adv. Sci..

[6] Martel J., Chang S.H., Ko Y.F., Hwang T.L., Young J.D., Ojcius D.M. (2022). Gut barrier disruption and chronic disease. Trends Endocrinol. Metab..

[7] Cani P.D., Jordan B.F. (2018). Gut microbiota-mediated inflammation in obesity: A link with gastrointestinal cancer. Nat. Rev. Gastroenterol. Hepatol..

[8] Gong Y., Xia W., Wen X., Lyu W., Xiao Y., Yang H., Zou X. (2020). Early inoculation with caecal fermentation broth alters small intestine morphology, gene expression of tight junction proteins in the ileum, and the caecal metabolomic profiling of broilers. J. Anim. Sci. Biotechnol..

[9] Carloni S., Bertocchi A., Mancinelli S., Bellini M., Erreni M., Borreca A., Braga D., Giugliano S., Mozzarelli A.M., Manganaro D. (2021). Identification of a choroid plexus vascular barrier closing during intestinal inflammation. Science.

[10] Chen H., Wang C., Bai J., Song J., Bu L., Liang M., Suo H. (2023). Targeting microbiota to alleviate the harm caused by sleep deprivation. Microbiol. Res..

[11] Gao T., Wang Z., Dong Y., Cao J., Lin R., Wang X., Yu Z., Chen Y. (2019). Role of melatonin in sleep deprivation-induced intestinal barrier dysfunction in mice. J. Pineal Res..

[12] Horowitz A., Chanez-Paredes S.D., Haest X., Turner J.R. (2023). Paracellular permeability and tight junction regulation in gut health and disease. Nat. Rev. Gastroenterol. Hepatol..

[13] Duan J., Matute J.D., Unger L.W., Hanley T., Schnell A., Lin X., Krupka N., Griebel P., Lambden C., Sit B. (2023). Endoplasmic reticulum stress in the intestinal epithelium initiates purine metabolite synthesis and promotes Th17 cell differentiation in the gut. Immunity.

[14] Pontarollo G., Kollar B., Mann A., Khuu M.P., Kiouptsi K., Bayer F., Brandão I., Zinina V.V., Hahlbrock J., Malinarich F. (2023). Commensal bacteria weaken the intestinal barrier by suppressing epithelial neuropilin-1 and Hedgehog signaling. Nat. Metab..

[15] Doré E., Joly-Beauparlant C., Morozumi S., Mathieu A., Lévesque T., Allaeys I., Duchez A.C., Cloutier N., Leclercq M., Bodein A. (2022). The interaction of secreted phospholipase A2-IIA with the microbiota alters its lipidome and promotes inflammation. JCI Insight.

[16] Lopez-Siles M., Duncan S.H., Garcia-Gil L.J., Martinez-Medina M. (2017). Faecalibacterium prausnitzii: From microbiology to diagnostics and prognostics. ISME J..

[17] Lopez-Siles M., Martinez-Medina M., Abellà C., Busquets D., Sabat-Mir M., Duncan S.H., Aldeguer X., Flint H.J., Garcia-Gil L.J. (2015). Mucosa-associated Faecalibacterium prausnitzii phylotype richness is reduced in patients with inflammatory bowel disease. Appl. Environ. Microbiol..

[18] Chen H., Ou R., Tang N., Su W., Yang R., Yu X., Zhang G., Jiao J., Zhou X. (2023). Alternation of the gut microbiota in irritable bowel syndrome: An integrated analysis based on multicenter amplicon sequencing data. J. Transl. Med..

[19] Ueda A., Shinkai S., Shiroma H., Taniguchi Y., Tsuchida S., Kariya T., Kawahara T., Kobayashi Y., Kohda N., Ushida K. (2021). Identification of Faecalibacterium prausnitzii strains for gut microbiome-based intervention in Alzheimer’s-type dementia. Cell Rep. Med..

[20] Khan M.T., Dwibedi C., Sundh D., Pradhan M., Kraft J.D., Caesar R., Tremaroli V., Lorentzon M., Bäckhed F. (2023). Synergy and oxygen adaptation for development of next-generation probiotics. Nature.

[21] Zhang L., Liu C., Jiang Q., Yin Y. (2021). Butyrate in Energy Metabolism: There Is Still More to Learn. Trends Endocrinol. Metab..

[22] Liang L., Liu L., Zhou W., Yang C., Mai G., Li H., Chen Y. (2022). Gut microbiota-derived butyrate regulates gut mucus barrier repair by activating the macrophage/WNT/ERK signaling pathway. Clin. Sci..

[23] Song Q., Cheng S.W., Zou J., Li K.S.L., Cheng H., Wai Lau D.T., Han Q., Yang X., Shaw P.C., Zuo Z. (2024). Role of gut microbiota on regulation potential of Dendrobium officinale Kimura & Migo in metabolic syndrome: In-vitro fermentation screening and in-vivo verification in db/db mice. J. Ethnopharmacol..

[24] Li H.B., Xu M.L., Xu X.D., Tang Y.Y., Jiang H.L., Li L., Xia W.J., Cui N., Bai J., Dai Z.M. (2022). Faecalibacterium prausnitzii Attenuates CKD via Butyrate-Renal GPR43 Axis. Circ. Res..

[25] Pan W., Zhao J., Wu J., Xu D., Meng X., Jiang P., Shi H., Ge X., Yang X., Hu M. (2023). Dimethyl itaconate ameliorates cognitive impairment induced by a high-fat diet via the gut-brain axis in mice. Microbiome.

[26] Ma L., Shen Q., Lyu W., Lv L., Wang W., Yu M., Yang H., Tao S., Xiao Y. (2022). Clostridium butyricum and Its Derived Extracellular Vesicles Modulate Gut Homeostasis and Ameliorate Acute Experimental Colitis. Microbiol. Spectr..

[27] Wu J., Lin Z., Wang X., Zhao Y., Zhao J., Liu H., Johnston L.J., Lu L., Ma X. (2022). Limosilactobacillus reuteri SLZX19-12 Protects the Colon from Infection by Enhancing Stability of the Gut Microbiota and Barrier Integrity and Reducing Inflammation. Microbiol. Spectr..

[28] Palmer C.A., Bower J.L., Cho K.W., Clementi M.A., Lau S., Oosterhoff B., Alfano C.A. (2023). Sleep loss and emotion: A systematic review and meta-analysis of over 50 years of experimental research. Psychol. Bull..

[29] Hyun M.K., Baek Y., Lee S. (2019). Association between digestive symptoms and sleep disturbance: A cross-sectional community-based study. BMC Gastroenterol..

[30] Sang D., Lin K., Yang Y., Ran G., Li B., Chen C., Li Q., Ma Y., Lu L., Cui X.Y. (2023). Prolonged sleep deprivation induces a cytokine-storm-like syndrome in mammals. Cell.

[31] Touch S., Godefroy E., Rolhion N., Danne C., Oeuvray C., Straube M., Galbert C., Brot L., Alonso Salgueiro I., Chadi S. (2022). Human CD4+CD8α+ Tregs induced by Faecalibacterium prausnitzii protect against intestinal inflammation. JCI Insight.

[32] Li N., Tan S., Wang Y., Deng J., Wang N., Zhu S., Tian W., Xu J., Wang Q. (2023). Akkermansia muciniphila supplementation prevents cognitive impairment in sleep-deprived mice by modulating microglial engulfment of synapses. Gut Microbes..

[33] Furter M., Sellin M.E., Hansson G.C., Hardt W.D. (2019). Mucus Architecture and Near-Surface Swimming Affect Distinct Salmonella Typhimurium Infection Patterns along the Murine Intestinal Tract. Cell Rep..

[34] McLoughlin K., Schluter J., Rakoff-Nahoum S., Smith A.L., Foster K.R. (2016). Host Selection of Microbiota via Differential Adhesion. Cell Host Microbe..

[35] Birchenough G.M.H., Schroeder B.O., Sharba S., Arike L., Recktenwald C.V., Puértolas-Balint F., Subramani M.V., Hansson K.T., Yilmaz B., Lindén S.K. (2023). Muc2-dependent microbial colonization of the jejunal mucus layer is diet sensitive and confers local resistance to enteric pathogen infection. Cell Rep..

[36] Turpin W., Lee S.H., Raygoza Garay J.A., Madsen K.L., Meddings J.B., Bedrani L., Power N., Espin-Garcia O., Xu W., Smith M.I. (2020). Increased Intestinal Permeability Is Associated with Later Development of Crohn’s Disease. Gastroenterology.

[37] Pedersen T.K., Brown E.M., Plichta D.R., Johansen J., Twardus S.W., Delorey T.M., Lau H., Vlamakis H., Moon J.J., Xavier R.J. (2022). The CD4(+) T cell response to a commensal-derived epitope transitions from a tolerant to an inflammatory state in Crohn’s disease. Immunity.

[38] Carlsson A.H., Yakymenko O., Olivier I., Håkansson F., Postma E., Keita A.V., Söderholm J.D. (2013). Faecalibacterium prausnitzii supernatant improves intestinal barrier function in mice DSS colitis. Scand. J. Gastroenterol..

[39] Martin R.M., Bachman M.A. (2018). Colonization, Infection, and the Accessory Genome of Klebsiella pneumoniae. Front. Cell Infect. Microbiol..

[40] Zhang Q., Su X., Zhang C., Chen W., Wang Y., Yang X., Liu D., Zhang Y., Yang R. (2023). Klebsiella pneumoniae Induces Inflammatory Bowel Disease Through Caspase-11-Mediated IL18 in the Gut Epithelial Cells. Cell Mol. Gastroenterol. Hepatol..

[41] Wasfi R., Hamed S.M., Amer M.A., Fahmy L.I. (2020). Proteus mirabilis Biofilm: Development and Therapeutic Strategies. Front. Cell Infect. Microbiol..

[42] Zhou X., Lu J., Wei K., Wei J., Tian P., Yue M., Wang Y., Hong D., Li F., Wang B. (2021). Neuroprotective Effect of Ceftriaxone on MPTP-Induced Parkinson’s Disease Mouse Model by Regulating Inflammation and Intestinal Microbiota. Oxid. Med. Cell Longev..

[43] Howden B.P., Giulieri S.G., Wong Fok Lung T., Baines S.L., Sharkey L.K., Lee J.Y.H., Hachani A., Monk I.R., Stinear T.P. (2023). Staphylococcus aureus host interactions and adaptation. Nat. Rev. Microbiol..

[44] Cani P.D., Depommier C., Derrien M., Everard A., de Vos W.M. (2022). Akkermansia muciniphila: Paradigm for next-generation beneficial microorganisms. Nat. Rev. Gastroenterol. Hepatol..

[45] Lukovac S., Belzer C., Pellis L., Keijser B.J., de Vos W.M., Montijn R.C., Roeselers G. (2014). Differential modulation by Akkermansia muciniphila and Faecalibacterium prausnitzii of host peripheral lipid metabolism and histone acetylation in mouse gut organoids. mBio.

[46] Liu J., Kang R., Tang D. (2024). Lipopolysaccharide delivery systems in innate immunity. Trends Immunol..

[47] Lai J.L., Liu Y.H., Liu C., Qi M.P., Liu R.N., Zhu X.F., Zhou Q.G., Chen Y.Y., Guo A.Z., Hu C.M. (2017). Indirubin Inhibits LPS-Induced Inflammation via TLR4 Abrogation Mediated by the NF-kB and MAPK Signaling Pathways. Inflammation.

[48] Ju M., Liu B., He H., Gu Z., Liu Y., Su Y., Zhu D., Cang J., Luo Z. (2018). MicroRNA-27a alleviates LPS-induced acute lung injury in mice via inhibiting inflammation and apoptosis through modulating TLR4/MyD88/NF-κB pathway. Cell Cycle.

[49] Zhou L., Zhang M., Wang Y., Dorfman R.G., Liu H., Yu T., Chen X., Tang D., Xu L., Yin Y. (2018). Faecalibacterium prausnitzii Produces Butyrate to Maintain Th17/Treg Balance and to Ameliorate Colorectal Colitis by Inhibiting Histone Deacetylase 1. Inflamm Bowel Dis..

[50] Gao T., Wang Z., Dong Y., Cao J., Chen Y. (2021). Melatonin-Mediated Colonic Microbiota Metabolite Butyrate Prevents Acute Sleep Deprivation-Induced Colitis in Mice. Int. J. Mol. Sci..

[51] Singh V., Lee G., Son H., Koh H., Kim E.S., Unno T., Shin J.H. (2022). Butyrate producers, “The Sentinel of Gut”: Their intestinal significance with and beyond butyrate, and prospective use as microbial therapeutics. Front. Microbiol..

[52] Fu X., Liu Z., Zhu C., Mou H., Kong Q. (2019). Nondigestible carbohydrates, butyrate, and butyrate-producing bacteria. Crit. Rev. Food Sci. Nutr..

[53] Recharla N., Geesala R., Shi X.Z. (2023). Gut Microbial Metabolite Butyrate and Its Therapeutic Role in Inflammatory Bowel Disease: A Literature Review. Nutrients.

[54] Chen Y., Liu Y., Wang Y., Chen X., Wang C., Chen X., Yuan X., Liu L., Yang J., Zhou X. (2022). Prevotellaceae produces butyrate to alleviate PD-1/PD-L1 inhibitor-related cardiotoxicity via PPARα-CYP4X1 axis in colonic macrophages. J. Exp. Clin. Cancer Res..

[55] Couto M.R., Gonçalves P., Magro F., Martel F. (2020). Microbiota-derived butyrate regulates intestinal inflammation: Focus on inflammatory bowel disease. Pharmacol. Res..

[56] Martín R., Rios-Covian D., Huillet E., Auger S., Khazaal S., Bermúdez-Humarán L.G., Sokol H., Chatel J.M., Langella P. (2023). Faecalibacterium: A bacterial genus with promising human health applications. FEMS Microbiol. Rev..

