# Supplementary material for: Faecalibacterium prausnitzii Supplementation Prevents Intestinal Barrier Injury and Gut Microflora Dysbiosis Induced by Sleep Deprivation
Source: Nutrients. 2024 Apr 9;16(8):1100. doi: 10.3390/nu16081100 (PMC11054126; doi:10.3390/nu16081100)
Supplement: Supplementary file 1 [file nutrients-16-01100-s001.zip › nutrients-2909408-supplementary.pdf]

Supplementary Material

Supplemental Table

Table S1 Primers for Real-time PCR

Gene name	Primer sequence	Produce size	Accession
F4/80	F: GCTGTGAGATTGTGGAAGCA	135	NM_001424712.1
	R: CTGTACCCACATGGCTGATG		
Tnf-α	F: CCCACACCGTCAGCCGATTT	200	NM_001278601.1
	R:GTCTAAGTACTTGGGCAGATTGACC		
Il-6	F: TAGTCCTTCCTACCCCAATTTCC	76	NM_031168.2
	R: TTGGTCCTTAGCCACTCCTTC		
Il-1β	F: GAAATGCCACCTTTTGACAGTG	116	NM_008361.4
	R: TGGATGCTCTCATCAGGACAG		
Il-10	F: GCTCTTACTGACTGGCATGAG	105	NM_010548.2
	R: CGCAGCTCTAGGAGCATGTG		
Mcp-1	F: CAGGTCCCTGTCATGCTTCT	250	NM_011333.3
	R: TCTGGACCCATTCCTTCTTG		
Claudin 1	F: GGGGACAACATCGTGACCG	100	NM_016674.4
	R: AGGAGTCGAAGACTTTGCACT		
Claudin 3	F: ACCAACTGCGTACAAGACGAG	78	NM_009902.4
	R: CAGAGCCGCCAACAGGAAA		
Zo-1	F: AAGAATATGGTCTTCGATTGGC	131	NM_009386.3
	R:ATTTTCTGTCACAGTACCATTTATCTTC		
Occludin	F: CAGCCTCGGTACAGCAGCAAT	102	NM_001360538.1
	R: ATAGTGGTCAGGGTCCGTCCTC		
Muc2	F: TGCTGCTGACGAGTGGTTGGTG	76	NM_023566.4
	R: CGGACGCTTGGTGGTGAGGC		
Bax	F: TGAAGACAGGGGCCTTTTTG	140	NM_007527.4
	R: AATTCGCCGGAGACACTCG		
Bcl-2	F: ATGCCTTTGTGGAACTATATGGC	120	NM_009741.5
	R: GGTATGCACCCAGAGTGATGC		
Gapdh	F: CCGAGAATGGGAAGCTTGTC	232	NM_001411843.1
	R: TTCTCGTGGTTCACACCCATC		
